# Supplementary material for: PrimPol-dependent single-stranded gap formation mediates homologous recombination at bulky DNA adducts
Source: Nat Commun. 2020 Nov 17;11:5863. doi: 10.1038/s41467-020-19570-7 (PMC7673990; doi:10.1038/s41467-020-19570-7)
Supplement: Supplementary file 1 — Supplemetary Information [file 41467_2020_19570_MOESM1_ESM.pdf]

Supplementary Information

PrimPol-dependent single-stranded gap formation mediates homologous recombination at bulky DNA adducts

Ann Liza Piberger, Akhil Bowry, Richard D W Kelly, Alexandra K Walker, Daniel Gonzalez-Acosta, Laura J Bailey, Aidan J Doherty, Juan Méndez, Joanna R Morris, Helen E Bryant, Eva Petermann

Supplementary Figures

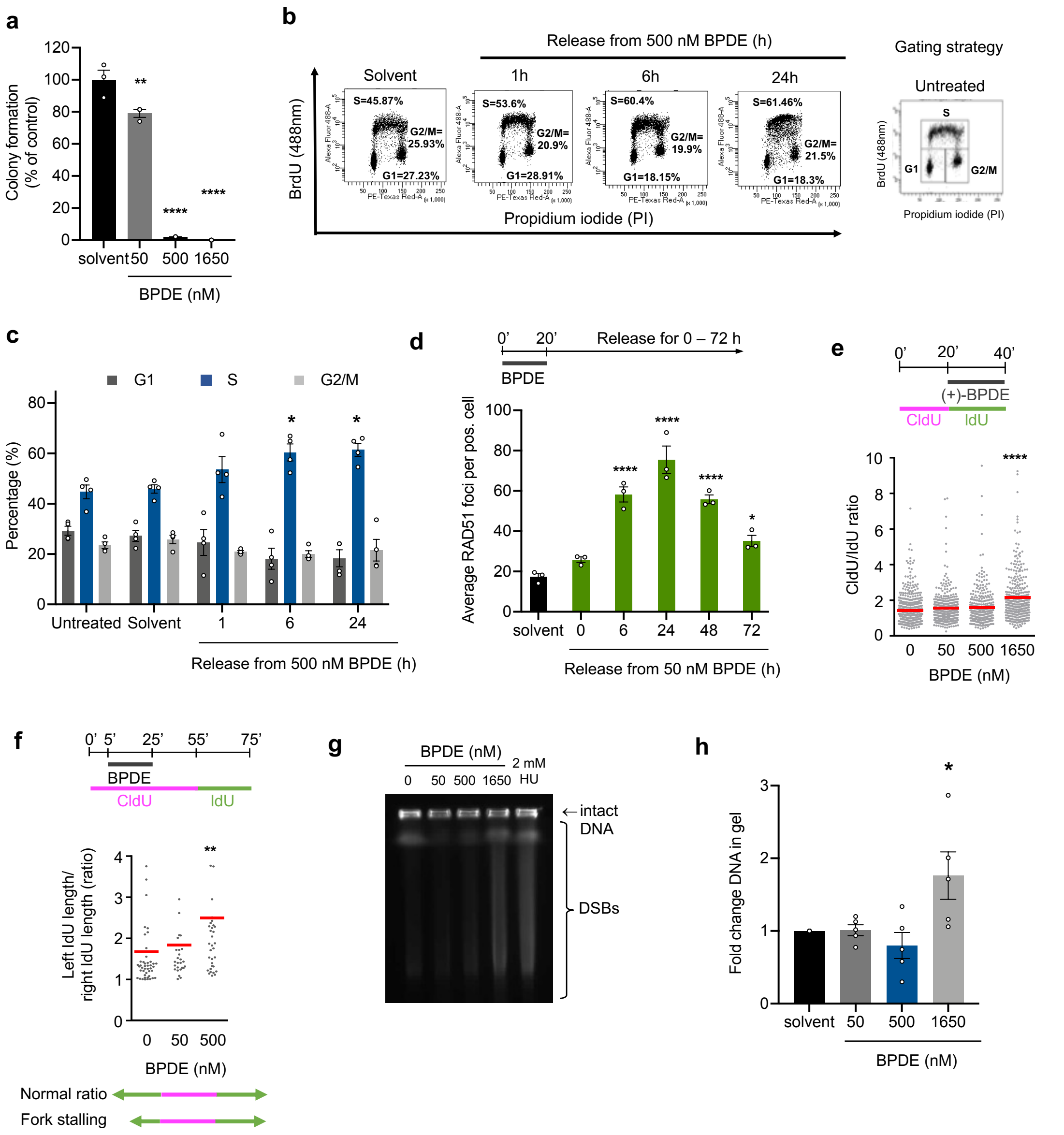

Supplementary Figure 1. Replication stress phenotypes induced by different BPDE concentrations.
(A) Colony survival of U2OS cells after release from 20 min BPDE. n=3 (B) Cell cycle distributions after release from solvent or 500 nM BPDE. Cells were labelled with BrdU for 30 min to label replicating cells. Right panel shows gating strategy. Release from solvent was for 1 h. (C) Quantification of cell cycle distributions after release from solvent or 500 nM BPDE as in B. n=4. (D) Numbers of RAD51 foci per RAD51 foci-positive (> 5 foci) cells after release from 50 nM BPDE. n=3 (E) CldU/IdU ratios after DNA fibre assay with BPDE present during the IdU pulse. Increased CldU/IdU ratios indicate BPDE-induced fork slowing. Lines: mean. Data from 3 repeats. (F) Fork asymmetry of bidirectional replication forks after DNA fibre assay with BPDE. Increased left IdU/right IdU ratios indicate fork stalling. Lines: mean. Data from 4 repeats. (G) Pulse-field gel electrophoresis (PFGE) to visualise DSBs in after 3 h release from BPDE. Treatment with 2 mM hydroxyurea (HU) for 48 h was used as a positive control. (H) Quantification of PFGE. DSBs were normalized to the total amount of DNA, then normalised to control. n= 5.

Source data are provided as a Source Data file. The means and SEM (bars) of independent experiments are shown. Asterisks indicate p-values compared to solvent control unless indicated otherwise (one-way ANOVA, * $p < 0.05$, ** $p < 0.01$, *** $p < 0.001$, **** $p < 0.0001$).

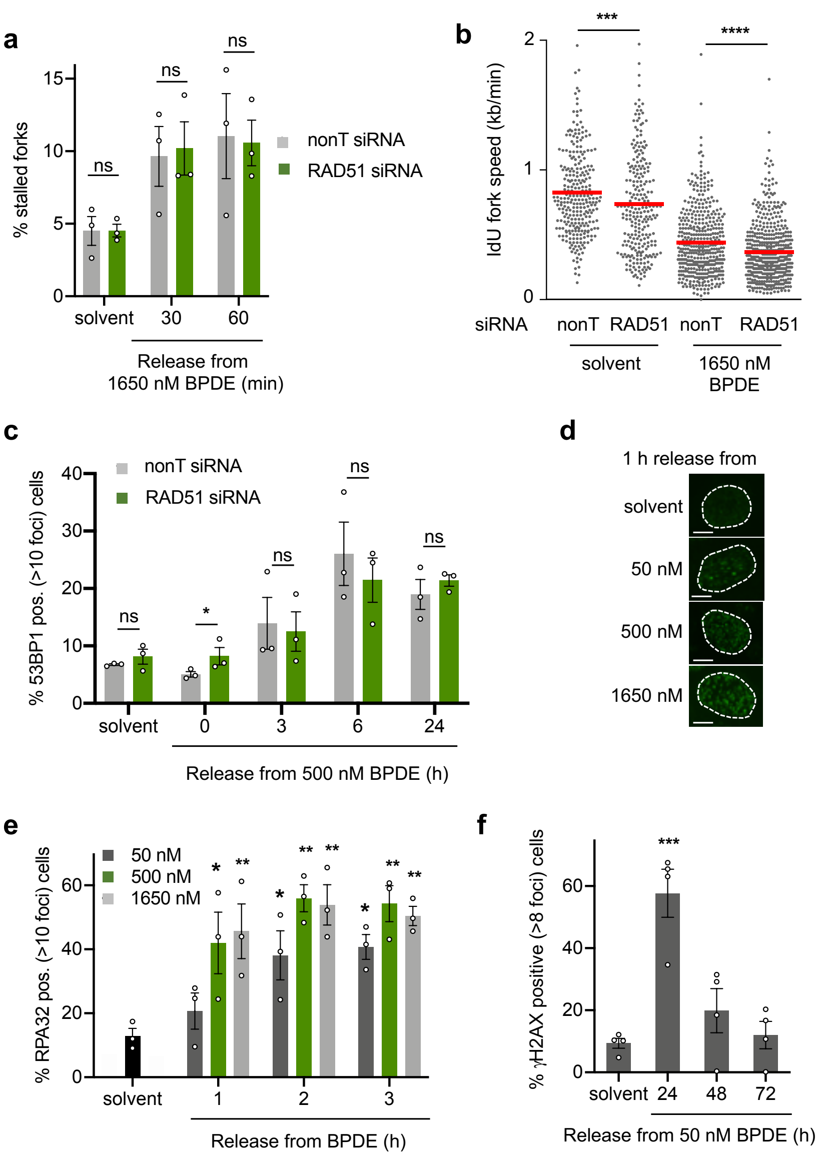

Supplementary Figure 2. Further investigation of potential roles for RAD51 in response to BPDE, and induction of RPA foci.
(A) Quantification of stalled forks after release from 1650 nM BPDE for 30 or 60 min in cells treated with non-targeting (nonT) or RAD51 siRNA. Release from solvent was 30 min. n=3 (B) Replication fork speeds in cells 30 min released from treatment with 1650 nM BPDE in presence or absence of RAD51 siRNA. Lines: mean. Data from 3 repeats. (C) Percentages of control- or RAD51-depleted U2OS cells with > 10 53BP1 foci after release from 500 nM BPDE. n=3. (D) Representative images of RPA foci after 1 h release from 20 min BPDE as indicated. Scale bars: 10 µm. (E) Percentages of U2OS cells with > 10 RPA foci after release from BPDE. Release from solvent was 1 h. n=3 (F) Percentages of U2OS cells with > 8 γH2AX foci after release from BPDE. Release from solvent was 24 h. n=4
Source data are provided as a Source Data file. The means and SEM (bars) of independent experiments are shown. Asterisks indicate p-values (one-sided student's t-test for A, C, one-sided Mann-Whitney for B, one-way ANOVA for E, F * $p < 0.05$, ** $p < 0.01$, *** $p < 0.001$, **** $p < 0.0001$).

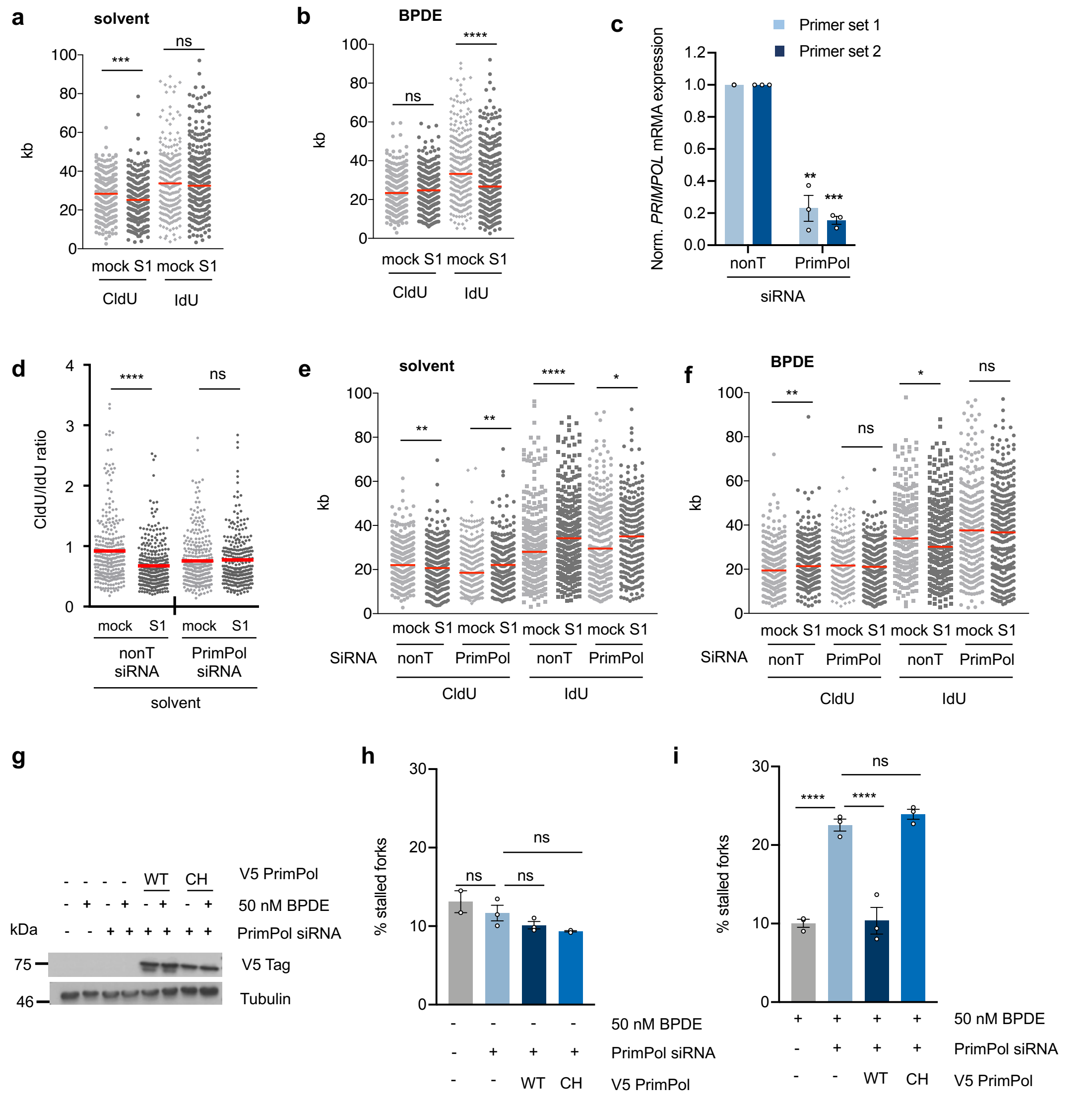

Supplementary Figure 3. BPDE induces single-stranded DNA gaps via PrimPol-dependent re-priming.

(A) Lengths of CldU and IdU tracks after S1-modified DNA fibre assay with solvent as in Figure 2C. Lines; median. Data from 3 repeats. (B) Lengths of CldU and IdU tracks after S1-modified DNA fibre assay with BPDE as in Figure 2C. Lines; median. Data from 3 repeats. (C) *PRIMPOL* mRNA quantification by qRT-PCR after 48 h siRNA transfection. *PRIMPOL* mRNA levels were normalised to *RPLP0* and control. n=3 (D) CldU/IdU ratios after S1-modified DNA fibre assay in cells treated with solvent as in Fig. 2G. Lines; mean. Data from 3 repeats. (E) Lengths of CldU and IdU tracks after S1-modified DNA fibre assay with solvent as in E. Lines; median. Data from 3 repeats. (F) Lengths of CldU and IdU tracks after S1-modified DNA fibre assay with BPDE as in Figure 3G. Lines; median. Data from 3 repeats. (G) Protein levels of V5-tagged WT or CH PrimPol and Tubulin (loading control) after release from 50 nM BPDE in presence of nonT (-) or PrimPol siRNA (H) Quantification of stalled forks after release from solvent in cells treated with nonT or PrimPol siRNA and expression constructs encoding GFP (-) or siRNA-resistant wild-type (WT) or primase-dead (CH) PrimPol. n=3. (I)

Quantification of stalled forks after release from 50 nM BPDE in cells treated as in F. n=3.
Source data are provided as a Source Data file. The means and SEM (bars) of independent experiments are shown. Asterisks indicate p-values compared to control (one-sided Mann-Whitney for A, B, D-F, one-sided Student's t-test for C and one-way ANOVA for H, I, * $p < 0.05$, ** $p < 0.01$, *** $p < 0.001$, **** $p < 0.0001$).

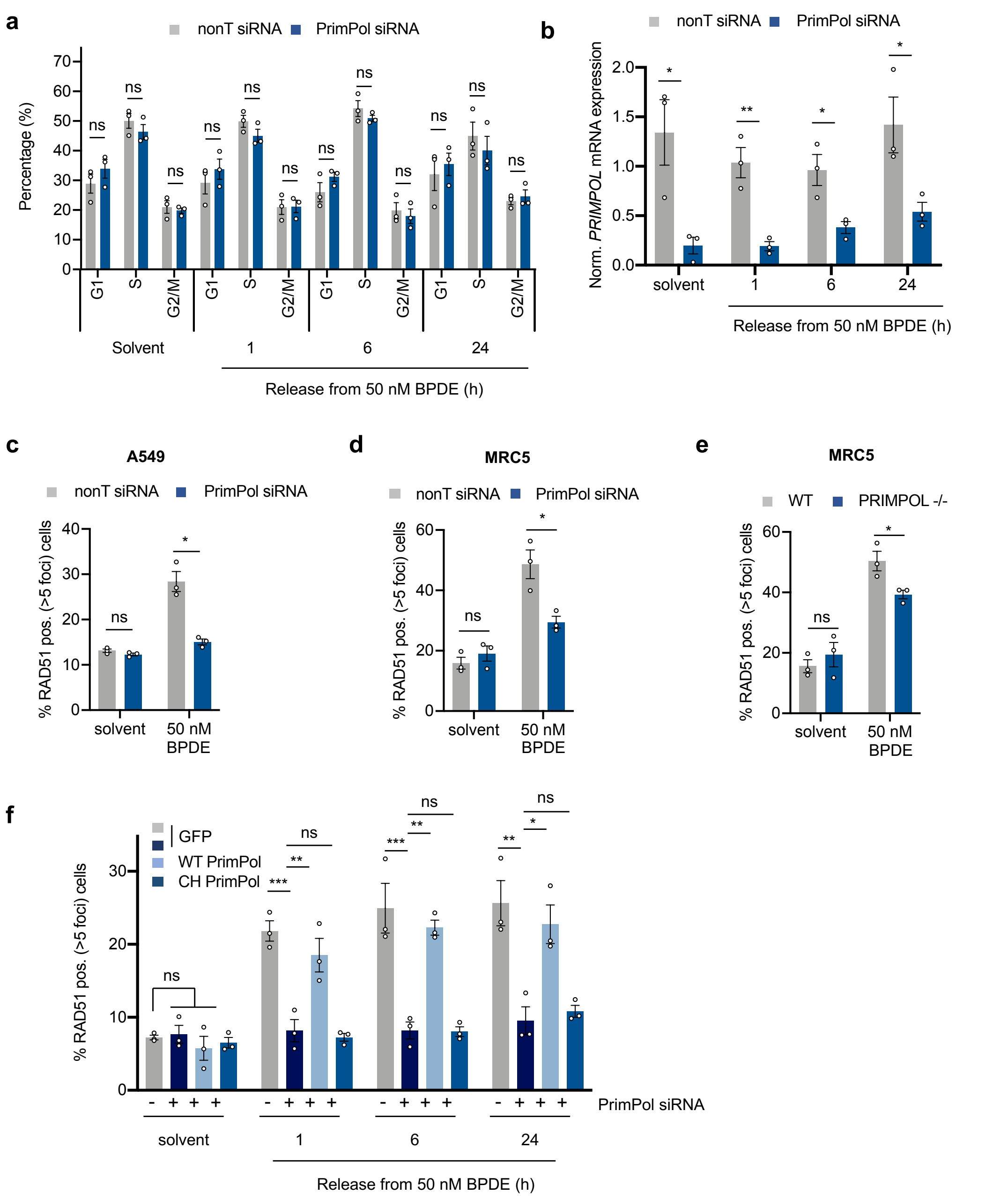

Supplementary Figure 4. Impact of PrimPol depletion on cell cycle distribution, and on RAD51 loading in response to BPDE in different cell lines.

(A) Quantification of cell cycle distributions after release from solvent (1 h) or 50 nM BPDE in presence of nonT or PrimPol siRNA, as in Fig. 3C. Cells were labelled with BrdU for 30 min to label replicating cells. Release from solvent was for 1 h. n=3. (B) *PRIMPOL* mRNA quantification by qRT-PCR after 48 h siRNA transfection and release from 20 min 50 nM BPDE as indicated. *PRIMPOL* mRNA levels were normalised to *RPLP0* and non-treated control. Release from solvent was for 1 h. n=3. (C, D) Percentages of A549 (C) or MRC5 (D) cells with >5 RAD51 foci after 1 h release from 50 nM BPDE, in presence of nonT or PrimPol siRNA. n=3. (E) Percentages of MRC5 WT or PrimPol $^{-/-}$ cells with >5 RAD51 foci after 1 h release from 50 nM BPDE. n=3. (F) Percentages of U2OS cells with >5 RAD51 foci after release from 50 nM

BPDE in presence of nonT (-) or PrimPol siRNA and expression constructs encoding GFP (-) or siRNA-resistant WT or CH PrimPol. Release from solvent was for 1 h. n=3.
Source data are provided as a Source Data file. The means and SEM (bars) of independent experiments are shown. Asterisks indicate p-values as indicated (one-sided student’s t-test for A - E or one-way ANOVA for F * $p < 0.05$, ** $p < 0.01$, *** $p < 0.001$).

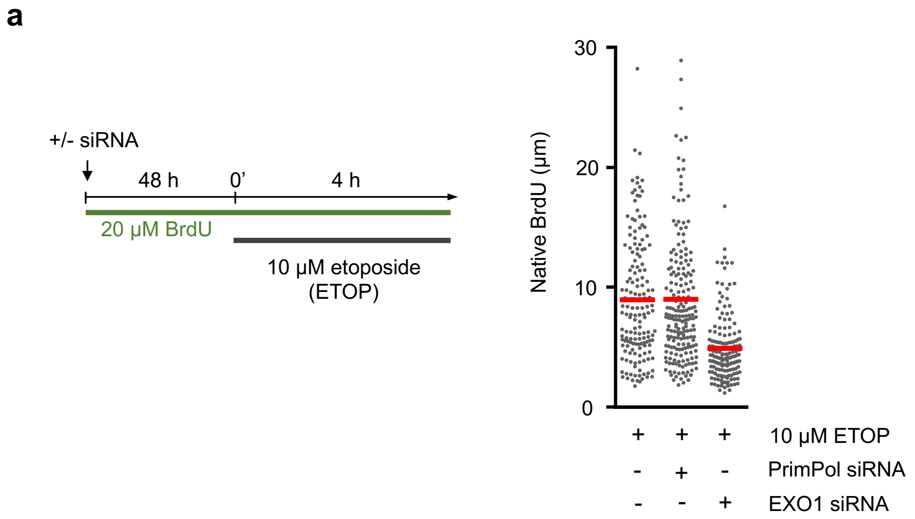

Supplementary Figure 5. ssDNA track lengths at etoposide-mediated DSBs are independent of PrimPol.
(A) SMART analysis of single-stranded DNA after treatment with etoposide (ETOP) as indicated, in presence of nonT (-), PrimPol, or EXO1 siRNA for 48 h. Lines; mean. Source data are provided as a Source Data file. Data from one independent experiment.

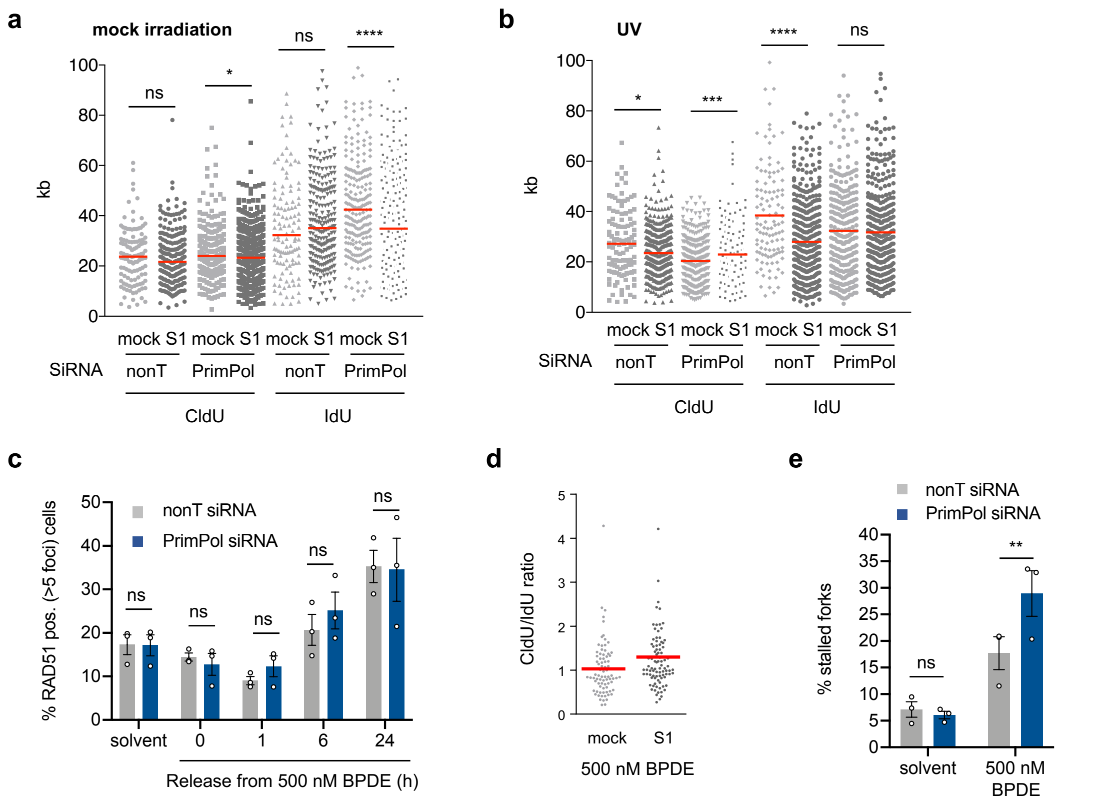

Supplementary Figure 6. PrimPol mediates RAD51 loading in response to bulky adducts, but not at DSBs induced by high BPDE concentrations.
(A) Lengths of CldU and IdU tracks after S1-modified DNA fibre assay with mock irradiation as in Figure 5B. Lines; median. Data from 4 repeats. (B) Lengths of CldU and IdU tracks after S1-modified DNA fibre assay with ultraviolet (UV) irradiation as in Figure 5B. Lines; median. Data from 4 repeats. (C) Percentages of cells with >5 foci after release from 500 nM BPDE for the times indicated, in presence of nonT or PrimPol siRNA. n=3 (D) CldU/IdU ratios after S1-modified DNA fibre assay in cells treated with 500 nM BPDE as in Fig. 2B. Lines: mean. Data from 2 repeats. (E) Quantification of stalled forks after release from 500 nM BPDE in presence of non-targeting (nonT) or PrimPol siRNA. Fibre labelling was according to Fig. 2F. n=3. Source data are provided as a Source Data file. The means and SEM (bars) of independent experiments are shown. Asterisks indicate p-values (one-sided Mann-Whitney for A, B, one-sided student's t-test for C, E * p < 0.05, ** p < 0.01, *** p < 0.001, **** p < 0.0001).

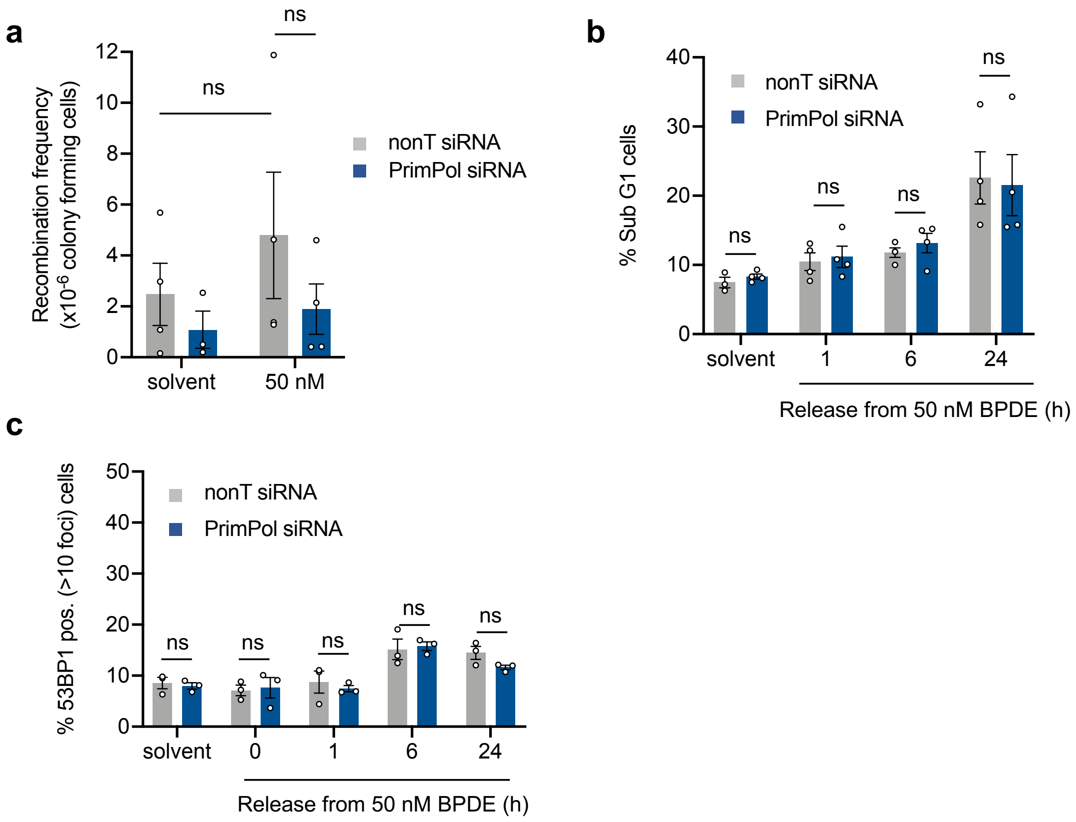

Supplementary Figure 7. Impact of PrimPol on recombination, cell death and DSB formation induced by bulky DNA adducts.
(A) Absolute recombination frequencies in SW480SN.3 cells induced by 50 nM BPDE in presence of nonT or PrimPol siRNA. n=4 (PrimPol siRNA with solvent n=3). (B) FACS analysis of percentages of sub-G1 cells after release from solvent or 50 nM BPDE in presence of nonT or PrimPol siRNA. Release from solvent was for 1 h. n=4 (nonT siRNA with solvent n=3). (C) Percentages of control- or PrimPol-depleted U2OS cells with > 10 53BP1 foci after release from 50 nM BPDE. n=3.
Source data are provided as a Source Data file. The means and SEM (bars) of independent experiments are shown. NS indicates non-significant p values (one-way ANOVA for A and one-sided student's t-test for B,C).

Supplementary Table 1. Sequences of qRT-PCR primers

Name	Sequence (5’ → 3’)
**PRIMPOL1 For**	CCGAGGTATCCCAGAGGTGA
**PRIMPOL1 Rev**	AATGCCCCACGTTGCTTTTC
**PRIMPOL2 For**	AAAAGCAACGTGGGGCATTC
**PRIMPOL2 Rev**	GGTGGTTCTTCTGGCTTGGA
**RPLP0 For (control)**	CAGATTGGCTACCCAACTGTT
**RPLP0 Rev (control)**	GGAAGGTGTAATCCGTCTCCAC
