# Supplementary material for: PrimPol-dependent single-stranded gap formation mediates homologous recombination at bulky DNA adducts
Source: Nat Commun. 2020 Nov 17;11:5863. doi: 10.1038/s41467-020-19570-7 (PMC7673990; doi:10.1038/s41467-020-19570-7)
Supplement: Supplementary file 3 — Reporting Summary [file 41467_2020_19570_MOESM3_ESM.pdf]

nature research

Corresponding author(s): Ann Liza Piberger and Eva Petermann

Last updated by author(s): 22/09/2020

Reporting Summary

Nature Research wishes to improve the reproducibility of the work that we publish. This form provides structure for consistency and transparency in reporting. For further information on Nature Research policies, see our Editorial Policies and the Editorial Policy Checklist.

Statistics

For all statistical analyses, confirm that the following items are present in the figure legend, table legend, main text, or Methods section.

n/a	Confirmed	
☐	☒	The exact sample size (*n*) for each experimental group/condition, given as a discrete number and unit of measurement
☐	☒	A statement on whether measurements were taken from distinct samples or whether the same sample was measured repeatedly
☐	☒	The statistical test(s) used AND whether they are one- or two-sided<br>*Only common tests should be described solely by name; describe more complex techniques in the Methods section.*
☒	☐	A description of all covariates tested
☐	☒	A description of any assumptions or corrections, such as tests of normality and adjustment for multiple comparisons
☐	☒	A full description of the statistical parameters including central tendency (e.g. means) or other basic estimates (e.g. regression coefficient) AND variation (e.g. standard deviation) or associated estimates of uncertainty (e.g. confidence intervals)
☐	☒	For null hypothesis testing, the test statistic (e.g. *F*, *t*, *r*) with confidence intervals, effect sizes, degrees of freedom and *P* value noted<br>*Give P values as exact values whenever suitable.*
☒	☐	For Bayesian analysis, information on the choice of priors and Markov chain Monte Carlo settings
☒	☐	For hierarchical and complex designs, identification of the appropriate level for tests and full reporting of outcomes
☒	☐	Estimates of effect sizes (e.g. Cohen's *d*, Pearson's *r*), indicating how they were calculated

Our web collection on statistics for biologists contains articles on many of the points above.

Software and code

Policy information about availability of computer code

Data collection	Volocity v6.1.1 (Perkin Elmer)<br>BD FacsDiva v8.0.1 (Becton Dickinson)
Data analysis	Image J version 1.50 (2016) and higher. Open source: http://rsbweb.nih.gov/ij/<br>BD FacsDiva v8.0.1 (Becton Dickinson)<br>Prism v6-v8 (Graphpad)<br>Microsoft Excel v16

For manuscripts utilizing custom algorithms or software that are central to the research but not yet described in published literature, software must be made available to editors and reviewers. We strongly encourage code deposition in a community repository (e.g. GitHub). See the Nature Research guidelines for submitting code & software for further information.

Data

Policy information about availability of data

All manuscripts must include a data availability statement. This statement should provide the following information, where applicable:

- Accession codes, unique identifiers, or web links for publicly available datasets
- A list of figures that have associated raw data
- A description of any restrictions on data availability

Raw data for this study mainly comprise microscopy images of the DNA fibre assay (Panels in Figures 1, 2, 4, 5, S1, S2, S3, S5, S6). These images are available from the authors upon request. Source data are provided as a Source Data file.

Field-specific reporting

Please select the one below that is the best fit for your research. If you are not sure, read the appropriate sections before making your selection.

- [x] Life sciences
- [ ] Behavioural & social sciences
- [ ] Ecological, evolutionary & environmental sciences

For a reference copy of the document with all sections, see nature.com/documents/nr-reporting-summary-flat.pdf

Life sciences study design

All studies must disclose on these points even when the disclosure is negative.

Sample size	No statistical tests were performed to determine sample size. Sample size was chosen by following the literature in the field.
Data exclusions	No data were excluded from the analysis, except for the time courses in Figure 1E and 1G, where data from two initial pilot experiments that used different time points were excluded for clarity.
Replication	To verify reproducibility, experiments were repeated 2, 3, 4 or 6 times as indicated in the figure legends. Attempts at replication were successful. Western blot for EXO1 depletion (Figure 4B) and SMART analysis after etoposide treatment (Supplementary Figure 5) were attempted just once as control experiments with clear results.
Randomization	Samples of cells (i.e. a group of cells in a dish, well, or flask) were allocated randomly to the treatments.
Blinding	Investigators were not blinded to group allocation during data collection and analysis. This was because we thought that blinding was not necessary to avoid bias, mainly because we are looking at fairly large effects and because results have been reproduced independently by different investigators in the group, including with using blinding.

Reporting for specific materials, systems and methods

We require information from authors about some types of materials, experimental systems and methods used in many studies. Here, indicate whether each material, system or method listed is relevant to your study. If you are not sure if a list item applies to your research, read the appropriate section before selecting a response.

Materials & experimental systems

n/a	Involved in the study	
[ ]	[x]	Antibodies
[ ]	[x]	Eukaryotic cell lines
[x]	[ ]	Palaeontology and archaeology
[x]	[ ]	Animals and other organisms
[x]	[ ]	Human research participants
[x]	[ ]	Clinical data
[x]	[ ]	Dual use research of concern

Methods

n/a	Involved in the study	
[x]	[ ]	ChIP-seq
[ ]	[x]	Flow cytometry
[x]	[ ]	MRI-based neuroimaging

Antibodies

Antibodies used

rabbit polyclonal anti-RAD51 (Abcam ab63801)
rabbit polyclonal anti-RAD51 (Calbiochem PC130)
mouse monoclonal anti-phospho-Histone H2AX (Ser139) (clone JBW301, Merck 05-636)
rabbit polyclonal anti-53BP1 (Bethyl A300-272A)
mouse monoclonal anti-RPA32 (clone RPA34-19, Merck NA18)
rat anti-BrdU (clone BU1/75, Abcam ab6326)
mouse monoclonal anti-BrdU (clone B44, Becton Dickinson 347580)
rabbit polyclonal anti-EXO1 (Bethyl, A302-640)
rabbit polyclonal anti-PRIMPOL (custom-made, described in ref 11)
rabbit polyclonal anti-SMC1 (custom-made, described in ref 58)
mouse monoclonal anti-V5 tag (clone R960-25, Invitrogen, R960-25)
mouse monoclonal anti-αTUBULIN (clone B512, Sigma T6074)
goat anti-mouse AlexaFluor 488 (Thermo Fisher, A-11001)
goat anti-rat AlexaFluor 594 (Thermo Fisher, A-21434)
anti-rabbit IgG AlexaFluor 594 (Thermo Fisher, A-11012)

Validation

rabbit polyclonal anti-RAD51 (Abcam ab63801)
Validated by us using Western blot and RAD51 siRNA depletion (Figure 1H). See also: Kondrashova, O., Topp, M., Nesic, K. et al. Methylation of all BRCA1 copies predicts response to the PARP inhibitor rucaparib in ovarian carcinoma. Nat Commun 9, 3970 (2018). Foci signal induced by IR and dependent on BRCA1 and RAD51C status.

rabbit polyclonal anti-RAD51 (Calbiochem PC130)
Validated in: King, O. et al. RAD51 Is a Selective DNA Repair Target to Radiosensitize Glioma Stem Cells. Stem Cell Rep 8,125-139 (2017). Foci signal inhibited by RAD51 inhibitors.

mouse monoclonal anti-phospho-Histone H2AX (Ser139) (clone JBW301, Merck 05-636)
Validated in: Frogkos, M. et al. H2AX Is Required for Cell Cycle Arrest via the p53/p21 Pathway. Mol Cell Biol 29, 2828-40 (2009). Signal is damage-induced, dependent on ATR activity and abrogated by H2AX siRNA depletion

rabbit polyclonal anti-53BP1 (Bethyl A300-272A)
Validated by Western blot and 53BP1 siRNA depletion in: Yamauchi, M., Shibata, A., Suzuki, K. et al. Regulation of pairing between broken DNA-containing chromatin regions by Ku80, DNA-PKcs, ATM, and 53BP1. Sci Rep 7, 41812 (2017).

mouse anti-RPA32 (clone RPA34-19, Merck NA18)
Validated by comparison with an antibody against phospho-S4/S8-RPA32. DNA damage-induced foci behave like phospho-S4/S8-RPA32 foci, and the band detected in Western blot shifts up when RPA32 is phosphorylated (Higgs MR. et al, Histone Methylation by SETD1A Protects Nascent DNA Through the Nucleosome Chaperone Activity of FANCD2. Mol Cell 71, 25-41 (2018))

rat anti-BrdU (clone BU1/75, Abcam ab6326)
Extensively validated as specific for BrdU-labelled S phase cells by Abcam https://www.abcam.com/brdu-antibody-bu175-icr1-proliferation-marker-ab6326.html
Shown by us as specific for CldU in DNA fibre assay: Kotsantis, P., Silva, L., Irmscher, S. et al. Increased global transcription activity as a mechanism of replication stress in cancer. Nat Commun 7, 13087 (2016).

mouse anti-BrdU (clone B44, Becton Dickinson 347580)
Shown by us as specific for BrdU-labelled S phase cells (Fig. 3C) and specific for IdU in DNA fibre assay: Kotsantis, P., Silva, L., Irmscher, S. et al. Increased global transcription activity as a mechanism of replication stress in cancer. Nat Commun 7, 13087 (2016).

rabbit anti-EXO1 (Bethyl, A302-640)
Shown by us as specific using EXO1 siRNA depletion and Western blotting (Figure 4B). See also a similar validation in: Przetocka et al., CtIP-Mediated Fork Protection Synergizes with BRCA1 to Suppress Genomic Instability upon DNA Replication Stress. Molecular Cell72, 568–582 (2018).

rabbit anti-PRIMPOL (described in ref 11)
Custom-made antibody. Shown by us as specific using PrimPol siRNA depletion and Western blotting, supported by qRT-PCR (Figure 2E, S3C). Detects V5-tagged ectopic PrimPol (Figure 2I). See also ref 11: Mourón S, et al. Repriming of DNA synthesis at stalled replication forks by human PrimPol. Nature structural & molecular biology 20, 1383-1389 10.1038/nsmb.2719 (2013).

rabbit anti-SMC1 (described in ref 59)
Custom-made antibody, validated using SMC1 siRNA in ref 59: Kojic A, et al. Distinct roles of cohesin-SA1 and cohesin-SA2 in 3D chromosome organization. Nature structural & molecular biology 25, 496-504 10.1038/s41594-018-0070-4 (2018).

mouse anti-V5 tag (clone R960-25, Invitrogen R960-25)
Shown by us as specific, detects V5-tagged PrimPol in Western blot (Figure 2I). See also validation on supplier website https://www.thermofisher.com/antibody/product/V5-Tag-Antibody-Monoclonal/R960-25

mouse anti-αTUBULIN (clone B512, Sigma T6074)
Used as loading control. Validated by Sigma-Aldrich using Western blot and by demonstrating antibody specificity through the use of multiple antibodies against target in IHC or ICC. https://www.sigmaaldrich.com/catalog/product/sigma/t6074?lang=en&region=GB&gclid=CjwKCAjw_qb3BRAVEiwAvwq6Vqv00IcIhxYBxMLzFCc7KcxeEWyq9CArdNN6RM2bYIrcyQhYCPpaPRoC78MQAvD_BwE

goat anti-mouse AlexaFluor 488 (Thermo Fisher, A-11001)
goat anti-rat AlexaFluor 594 (Thermo Fisher, A-21434)
anti-rabbit IgG AlexaFluor 594 (Thermo Fisher, A-11012)
These are widely used commercial secondary antibodies. For information see supplier's website:
https://www.thermofisher.com/antibody/product/Goat-anti-Mouse-IgG-H-L-Cross-Adsorbed-Secondary-Antibody-Polyclonal/A-11001
https://www.thermofisher.com/antibody/product/Goat-anti-Rat-IgG-H-L-Cross-Adsorbed-Secondary-Antibody-Polyclonal/A-21434
https://www.thermofisher.com/antibody/product/Goat-anti-Rabbit-IgG-H-L-Cross-Adsorbed-Secondary-Antibody-Polyclonal/A-11012

Eukaryotic cell lines

Policy information about cell lines

Cell line source(s)	U2OS (ATCC), MRC5 (ATCC), A549 (ATCC), MRC5 WT and PrimPol -/- (Aidan J. Doherty lab), SW480SN.3 (Helen E. Bryant lab), U2OS carrying doxycycline (Dox)-inducible PrimPol shRNA (Juan Mendez lab).
Authentication	The following cell lines were authenticated by 17-locus STR profiling through the ATCC Cell line Authentication Service: U2OS (100% match), A549 (100% match), MRC5 (87% match), U2OS shPrimPol (100% match). MRC5 WT, MRC5 PrimPol-/- and SW480SN.3 cell lines were not authenticated.

Mycoplasma contamination	All cell lines were tested for mycoplasma contamination using DAPI staining for cytoplasmic DNA. All cell lines tested mycoplasma negative.
Commonly misidentified lines (See ICLAC register)	None

Flow Cytometry

Plots

Confirm that:

☒ The axis labels state the marker and fluorochrome used (e.g. CD4-FITC).

☒ The axis scales are clearly visible. Include numbers along axes only for bottom left plot of group (a 'group' is an analysis of identical markers).

☒ All plots are contour plots with outliers or pseudocolor plots.

☒ A numerical value for number of cells or percentage (with statistics) is provided.

Methodology

Sample preparation	U2OS cells were transfected with siRNA for 48 h followed by treatment with 50 nM BPDE and release into fresh DMEM. Cells were incubated with 100 µM BrdU for the last 30 min of the indicated release times and then fixed in 70% ethanol overnight. Cells were incubated in 2 M HCl/0.1mg/ml pepsin for 30 min. Samples were washed in PBS before incubation with mouse anti-BrdU (B44, Becton Dickinson 347580, 1:200) for 1 h and subsequently with anti-mouse IgG AlexaFluor 488 (ThermoFisher) for 1 h. Finally, cells were incubated with 10 µg/ml propidium iodide and 25 µg/ml RNaseA for 30 min.
Instrument	BD LSR Fortessa X20
Software	BD FacsDiva v8.0.1 (Becton Dickinson)
Cell population abundance	Not applicable- Flow cytometry analysis was performed on a single cell line.
Gating strategy	FCS/SSC gating was used to excluded cell debris and Live/dead cell exclusion was performed using propidium iodide (PI). Gating strategy for cell cycle profiles are provided in Supplementary Figure 1B.

☒ Tick this box to confirm that a figure exemplifying the gating strategy is provided in the Supplementary Information.
